# Monogenic mutations in four cases of neonatal-onset watery diarrhea and a mutation review in East Asia

**DOI:** 10.1186/s13023-021-01995-y

**Published:** 2021-09-09

**Authors:** Weihui Yan, Yongtao Xiao, Yunyi Zhang, Yijing Tao, Yi Cao, Kunhui Liu, Wei Cai, Ying Wang

**Affiliations:** 1 Division of Pediatric Gastroenterology and Nutrition, Xinhua Hospital Affiliated To Shanghai Jiao Tong University School of Medicine, Shanghai, People’s Republic of China; 2 Shanghai Institute for Pediatric Research, Shanghai, People’s Republic of China; 3 Department of Pediatric Surgery, Xinhua Hospital Affiliated To Shanghai Jiao Tong University School of Medicine, Shanghai, People’s Republic of China; 4 Shanghai Key Laboratory of Pediatric Gastroenterology and Nutrition, Shanghai, People’s Republic of China; 5 Innovative Research Team of High-Level Local Universities in Shanghai, Shanghai, People’s Republic of China; 6 Xinhua Hospital Affiliated To Shanghai Jiao Tong University School of Medicine, No.1665, Kong Jiang Road, Yangpu, Shanghai, 200092 People’s Republic of China

**Keywords:** Microvillus inclusion disease, Congenital chloride diarrhea, Congenital tufting enteropathy, Neonatal-onset diarrhea, Whole-exome sequencing, Monogenetic mutation

## Abstract

**Background:**

Infants with neonatal-onset diarrhea present with intractable diarrhea in the first few weeks of life. A monogenic mutation is one of the disease etiologies and the use of next-generation sequencing (NGS) has made it possible to screen patients for their mutations.

**Main body:**

We retrospectively reviewed the clinical data of four children from unrelated families, who presented with neonatal-onset, chronic, watery, non-bloody diarrhea. After genetic whole-exome sequencing, novel mutations were identified in the *EPCAM* gene of two children. Congenital chloride diarrhea was diagnosed in one case, which was associated with an *SLC26A3* mutation, in which the patient presented with watery diarrhea, malnutrition, and hypochloremic alkalosis. Patient 4 was diagnosed with microvillus inclusion disease and possessed novel compound heterozygous mutations in the *MYO5B* gene. A review of the genetic variants of *SLC26A3* reported in East Asia revealed that c.269_270 dupAA (p.G91Kfs*3) is the most frequent *SLC26A3* mutation in China, compared with c.2063-1 G > T in Japan and Korea. *EPCAM* and *MYO5B* genetic variants were only sporadically reported in East Asia.

**Conclusion:**

This study expands our knowledge of the clinical manifestations and molecular genetics of neonatal-onset watery diarrhea. Early diagnosis could be achieved by genomic analysis in those infants whose histology features are not typical. The discovery of four novel mutations in the *EPCAM* gene and two novel mutations in the *MYO5B* gene provides further etiological evidence for the association of genetic mutations with neonatal-onset diarrhea. To date, c.269_270 dupAA is the most frequent *SLC26A3* mutation in China.

## Background

Neonatal-onset diarrhea is heterogeneous in its etiology, ranging from simple cow’s milk protein intolerance to life-threatening defects of membrane polarization [1]. One class of neonatal-onset diarrhea is congenital diarrhea and enteropathies (CODEs), which result from inherited disorders [2], and are distinguished from acquired diarrheas due to pathogen infection and food-protein allergies. These inherited disorders are rare and effective therapeutic options for these patients have not been established. CODEs are characterized by dramatic diarrhea in the first few weeks of life, which can potentially lead to intestinal failure (IF). The classification and diagnostic algorithm for CODEs have been reviewed by Thiagarajah [2].

Most CODEs are monogenic and can be classified into five major categories [1, 2]: (a) defects in epithelial nutrient and electrolyte transport, such as congenital chloride diarrhea (CCD) and glucose-galactose malabsorption; (b) epithelial enzymes and metabolism defects, such as congenital lactase deficiency and chylomicron retention disease; (c) defects in epithelial trafficking and polarity, such as microvillus inclusion disease (MVID) and congenital tufting enteropathy (CTE); (d) enteroendocrine cell dysfunction, such as enteric anendocrinosis; and (e) immune dysregulation-associated enteropathy, such as IPEX (immune dysregulation, polyendocrinopathy, enteropathy, X-linked) syndrome. The primary clinical manifestations of CCD, CTE, and MVID are high-volume watery diarrhea within the first several months of life [2].

Although many cases are strongly suspected to be CODEs, the specific etiology is not identified without genetic testing. A delayed diagnosis may result in irreversible complications and significant morbidity and mortality. Therefore, it is essential to identify these disorders early in order to prevent complications. Next-generation sequencing (NGS) has contributed to great advances in this field, by enabling investigations into the genetic basis of monogenic disorders causing CODEs and allowing for appropriate treatment to be initiated earlier.

In this study, we report four cases of patients who presented with neonatal-onset watery diarrhea and who carried monogenic mutations, including 2 CTEs, 1 MVID, and 1 CCD. We also review the genetic variants of solute carrier family 26 member 3 (*SLC26A3*), epithelial cell adhesion molecule (*EPCAM*), and myosin VB (*MYO5B*) that have been reported in East Asia.

## Results

### Clinical description

#### Patients

The clinical characteristics and histological profiles of the patients are summarized in Tables 1 and 2.

**Table 1:** The clinical characteristics of four patients with neonatal-onset watery diarrhea

		Patient 1	Patient 2	Patient 3	Patient 4
Gender		Male	Male	Male	Male
Birth weight (g)		2600	3340	3020	3300
Gestation age (weeks)		34	40	35 + 5	40 + 3
Age at onset of diarrhea		10 days	< 3 days	After birth	28 days
Family history		No	No	No	No
Age at admission to our hospital		23 months	17 days	Born in our hospital	42 days
Weight at admission (g)		6800	2700	3020	3518
Age at diagnosis		27 months	45 days	6 months	3 months
Weight at diagnosis (g)		8200	2955	6700	4134
Main symptoms	Diarrhea	Watery, 390–1230 g/d	Watery, > 10 /d	Watery, 3–5/d	Watery, > 10/d
	Vomiting	0–3/d	No	No	Yes
	Abdominal distention	Yes	No	PMA 24 weeks-4 days after birth	Yes
Laboratory examination (blood)	Age at examination	23 months	17 days	3 months	42 days
	C-reactive protein (mg/L)	< 8	8	< 8	< 8
	WBC (*10^9/L)	10.68	23	8.3	21.92
	ALT (U/L)	46	25	39	120
	Albumin (g/L)	42.3	33.4	40.5	37.6
	pH	7.35	7.29	7.59	7.14
	Chloride (mmol/L)	97	113	64	131
	Potassium (mmol/L)	4.4	3.4	2.63	2
	Sodium (mmol/L)	136	137	127.5	142
	Base excess (mmol/L)	0.2	− 15.3	24.6	–21.8
Complications	Sepsis	7 Episodes in 1 year	1 Episode in 2 months	No	Yes
	Vitamin D deficiency	Yes	ND	No	ND
	Liver dysfunction	Yes	Yes	No	Yes
	Seizure	Once	No	No	Recurrent
	Failure to thrive	Yes	Yes	Within 3 months	Yes
Enteral nutrition	Routes	Nasogastric/nasojejunal tube	Per os	Nasogastric tube	Nasogastric tube
	Formula	Peptide-based formula	AAF, eHF	eHF	AAF, eHF
Surgery		1st: Ileostomy; 2nd: Bishop Koop ileostomy; 3rd: stoma closure	No	Resection of 10 cm ileum, ileocecus and 1 cm colon; Bishop Koop ileostomy	No
PN weaned		No	No	Yes	No
Diagnosis for diarrhea		CTE	CTE	CCD	MVID
Follow-up		Died of blood transfusion reaction at 3.5 years of age	The child’s body weight was 3.3 kg on his first birthday	The child’s body weight was 8.5 kg with nasogastric feeding and supplementation with 8 ml/day 10% KCl at 8 months	Died after discharge

*EN* enteral nutrition, *PN* parenteral nutrition, *eHF* extensively hydrolyzed formula, *AAF* amino acid-based formula, *PMA* postmenopausal age, *CTE* congenital tufting enteropathy, *MVID* microvillus inclusion disease, *CCD* congenital chloride diarrhea, *ND* no data

**Table 2:** Pathology assessment of four patients with neonatal-onset watery diarrhea

	Age (procedure)	H&E staining	TEM assessment
Patient 1	11 months (endoscopy)	Rectum: active and chronic inflammation; cryptitis; 10 ± EOS/HPF	ND
	26 months (endoscopy)	Colon: chronic inflammation; tufts or teardrop appearance; eosinophils and plasma cells infiltration	ND
	27 months (surgery)	Stomach: chronic gastritis; submucosal edema, hyperemia Jejunum: villous blunting; tufts at villus tips; lymphocyte infiltration in the epithelial layer; prominent inflammatory cell infiltration in lamina propria Ileum: focal mucosal erosion and hyperemia; lymphocyte infiltration; tufts at villus tips Colon: tufts or teardrop appearance; hyperemia; edema	Jejunum: focal loss of microvilli and disorganized cellular junctions; Colon: focal loss of microvilli
	31 months (endoscopy)	Stomach: chronic gastritis Ileum: chronic inflammation; tufts at villus tips	ND
	34 months (surgery)	Colon: mucosal hyperemia, edema, and erosion; tufts or teardrop appearance	ND
	40 months (surgery and endoscopy)	Ileum: villous blunting or missing; tufts at villus tips; submucosal lymphoid hyperplasia Colon: chronic mucosal inflammation; tufts or teardrop appearance; inflammatory cell infiltration in lamina propria	ND
Patient 2	43 days (endoscopy)	Duodenum: villous atrophy; disorganized epithelium Ileum: villous atrophy; crowded epithelial cells Colon: chronic inflammation; villous atrophy	Ileum: flattened microvilli; swollen epithelial cells and slightly dilated endoplasmic reticulum; interstitial edema; infiltration of lymphocytes and plasma cells
Patient 3	4 days (surgery)	Ileum: hyperemia, edema, and lymphocyte aggregates; a proliferation of submucosal fibrous tissue Colon: hyperemia, edema, and lymphocyte aggregates; a proliferation of submucosal fibrous tissue	ND
Patient 4	71 days (endoscopy)	Duodenum: hyperemia, edema, and prominent lymphocyte infiltration; villous blunting Ileum: hyperemia, edema, and prominent lymphoid aggregates; villous blunting	Ileum: focal loss of microvilli; swollen epithelial cells; infiltration of interstitial plasma cells, lymphocytes, and eosinophils

*EOS* eosinophil, *HPF* high power field, *TEM* transmission electronic microscopy, *H&E* hematoxylin–eosin, *ND* no data

##### Patient 1

The boy was transferred to our hospital at the age of 23 months. He was born by cesarean delivery at 34 weeks gestation, with a birth weight of 2600 g. He has a healthy older sister who is 12 years old. The patient’s intractable diarrhea started on the 10th day after birth. He had to be hospitalized numerous times due to recurrent electrolyte disturbances, abdominal distension, vomiting, and sepsis. The treatments included antibiotics (piperacillin/tazobactam, cefmetazole, ceftazidime + metronidazole, or vancomycin), probiotics, smectite, and pancreatic enzymes, but there was no improvement in the patient’s condition. When the patient was admitted to our center, he presented with severe growth retardation, with a weight of 6.8 kg (Z score − 4.71) and a height of 70.5 cm (Z score − 5.35).

**Fig. 1:**
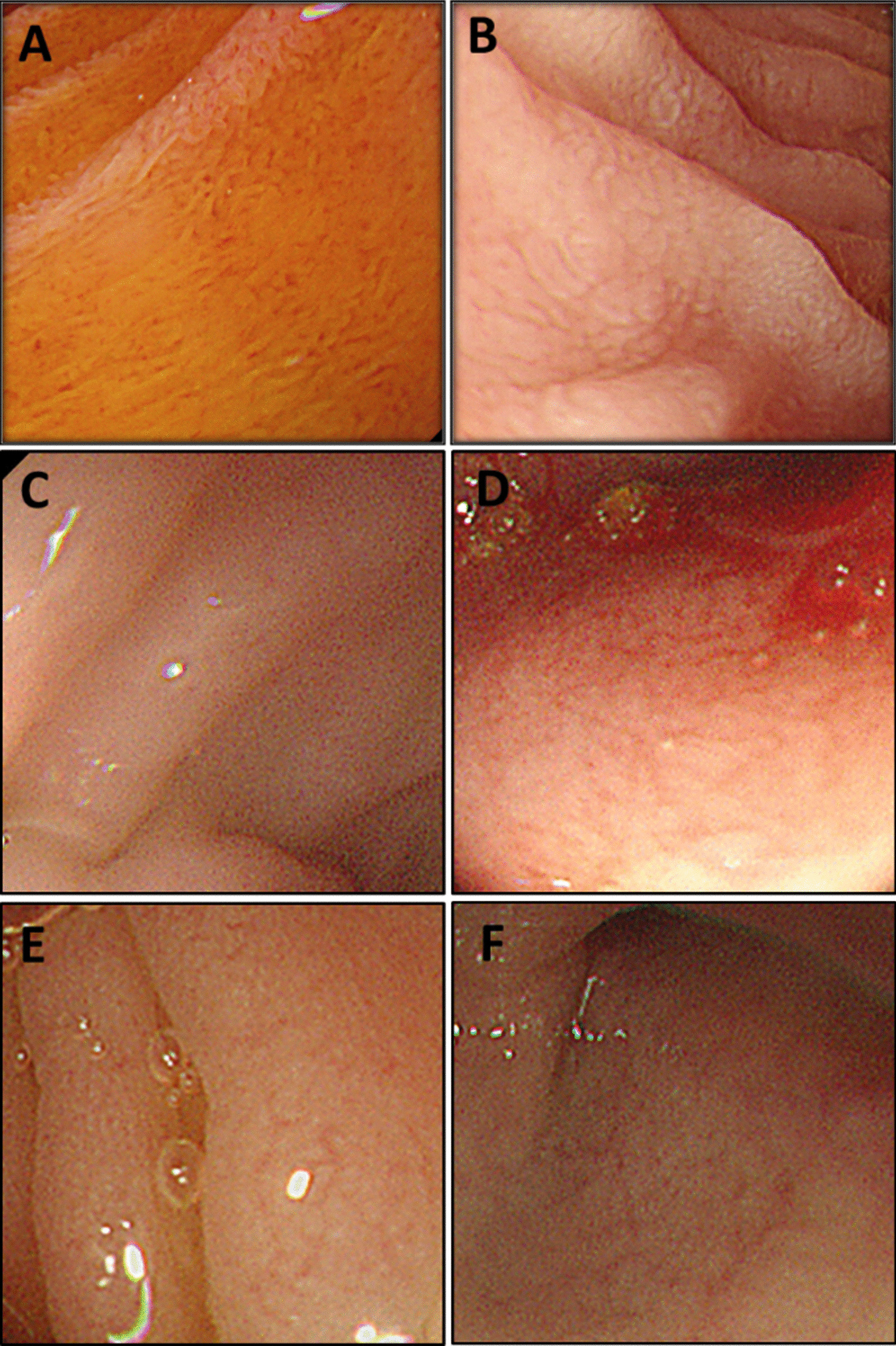
The endoscopic appearance of three patients. **A** Normal mucosa in the descending duodenum of a healthy 2-year-old child (Olympus GIF-H260 gastroscope). **B** Villous atrophy in the duodenal mucosa of Patient 1 at 26 months (Olympus GIF-H260 gastroscope). **C**, **D** No apparent gross abnormalities in the duodenum (**C**) or terminal ileum (**D**) of Patient 2 at 43 days (Olympus GIF-XP290N gastroscope). **E**, **F** No apparent gross abnormalities in the duodenum (**E**) or terminal ileum (**F**) of Patient 4 at 71 days (Olympus GIF-XP290N gastroscope)

Initial laboratory tests at admission revealed normal levels of serum liver enzymes, creatinine, immunoglobulins, thyroid hormones, negative autoimmune antibodies, and a normal complete blood cell count. The tests for enteric pathogens including Salmonella, Shigella and Cholera were negative. Colonoscopy examinations did not show any obvious abnormalities. Compared with a patient at the same age (Fig. 1A), an upper endoscopy revealed villous atrophy in the duodenal mucosa (Fig. 1B).

**Fig. 2:**
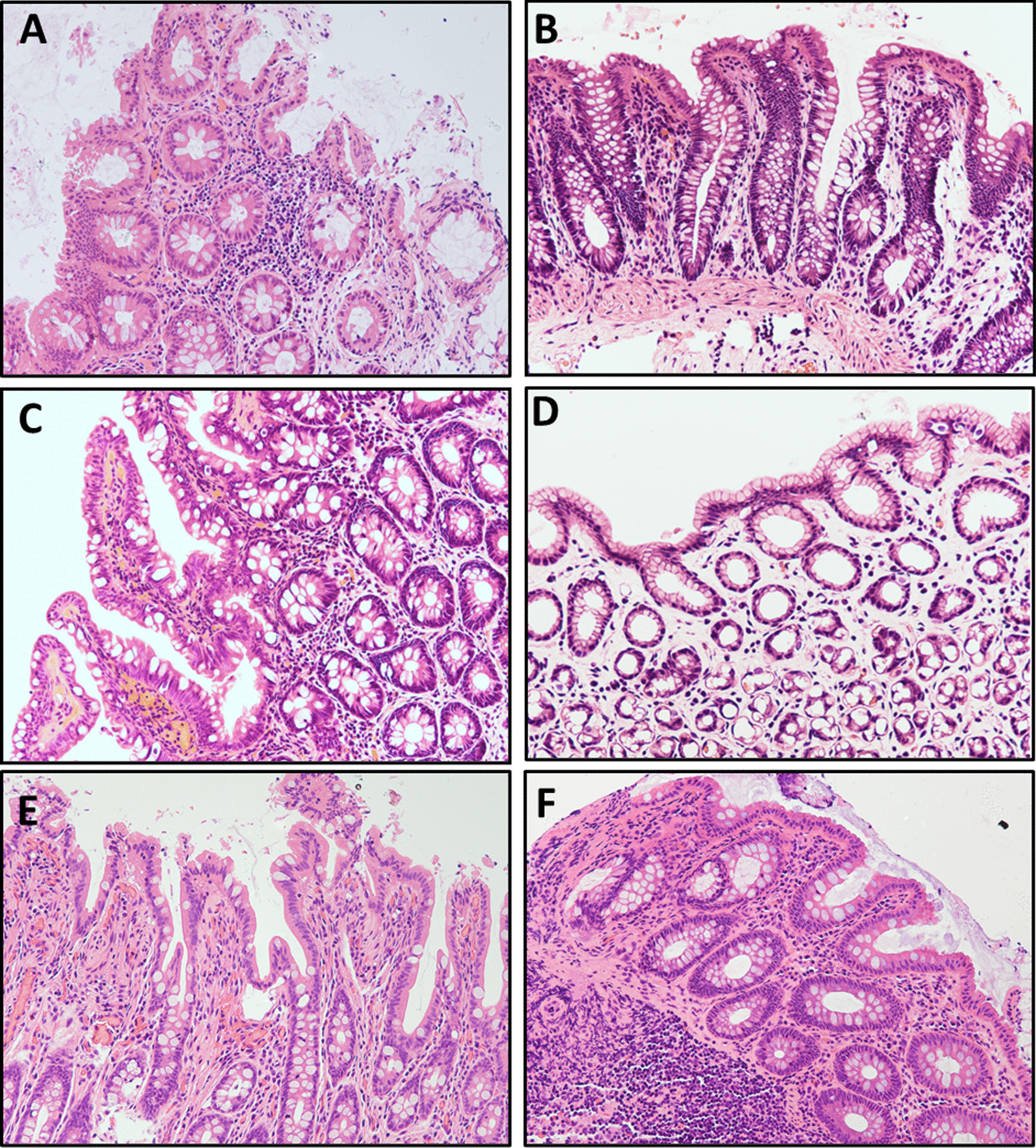
H&E staining of intestinal biopsy from Patient 1. **A** Chronic inflammation; tufts or teardrop appearance; infiltration of eosinophils and plasma cells in the colon at 26 months (200×). **B** Atrophic villi with crowded epithelial cells forming tufts in the jejunum biopsy at 27 months (200×). **C** Villus shortening, disorganized proliferating epithelial cells, focal tufting, and crypt hyperplasia in the ileum biopsy at 27 months (200×). **D** Tufts or teardrop appearance and crypt hyperplasia in the colon at 27 months (200×). **E** Villous blunting; tufts at villus tips in the ileum near the stoma at 40 months (200×). **F** Chronic mucosal inflammation; tufts or teardrop appearance; inflammatory cell infiltration in the lamina propria of the colon at 40 months (200×)

**Fig. 3:**
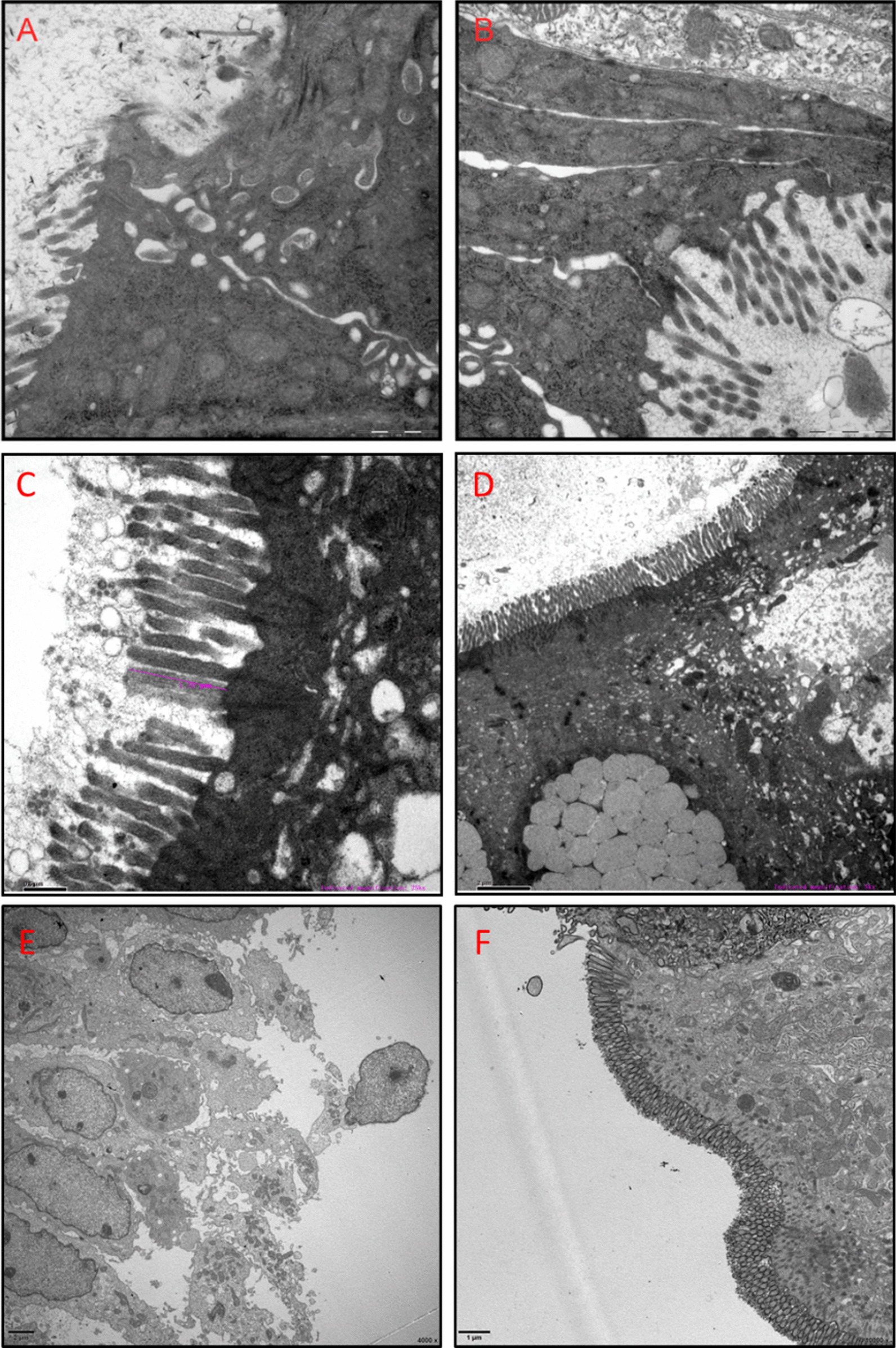
TEM scanning of intestinal tissue from three patients. **A**, **B** Focal loss of microvilli and disorganized cellular junctions in the jejunum of Patient 1 (**A**, **B**. 30,000×). **C**, **D** Flattened microvilli; swollen epithelial cells and slightly dilated endoplasmic reticulum in the ileum of Patient 2 (**C**. 25,000× ; **D**. 5000×). **E**, **F** Focal loss of microvilli; swollen epithelial cells in the ileum of Patient 4 (**E**. 4000× ; **F**. 10,000×)

The child’s stool output decreased to 390 g/day at the cessation of enteral nutrition (EN). After EN (peptide formula) was reintroduced, the stool output increased to 505–1230 g/day. He underwent 7 episodes of sepsis from Nov 2017 to Aug 2018. Because a dilated colon was detected by barium enema and a diagnosis of recurrent gut-origin sepsis was considered, a terminal ileostomy was performed to rest the colon. Hematoxylin–eosin staining of biopsied tissue showed flattening villi, crypt hyperplasia, disorganization of the surface epithelium, and tufts at the villus tips (Fig. 2). Transmission electron microscopy (TEM) revealed a decreased number of microvilli and disorganized cellular junctions (Fig. 3A, B).

The patient’s stool output fluctuated from 143 to 452 g daily after he underwent a Bishop Koop ileostomy at 34 months and received a fecal microbiota transplant (FMT). His nutritional status improved significantly as a result of nasojejunal feeding with peptide-based formula and parenteral nutrition (PN) support. The child’s body weight was 12.5 kg (Z score − 1.55) when he was 40 months old at the last discharge from our hospital. Unfortunately, he died of a severe reaction to a blood transfusion at the age of 3.5 years in a local hospital.

##### Patient 2

The boy was admitted to the neonatal intensive care unit (NICU) at 17 days old. He was born with a weight of 3340 g at 40 weeks gestation by vaginal delivery. He suffered from persistent, more than 10 per day, watery, non-bloody diarrhea stools within 3 days after birth.

**Fig. 4:**
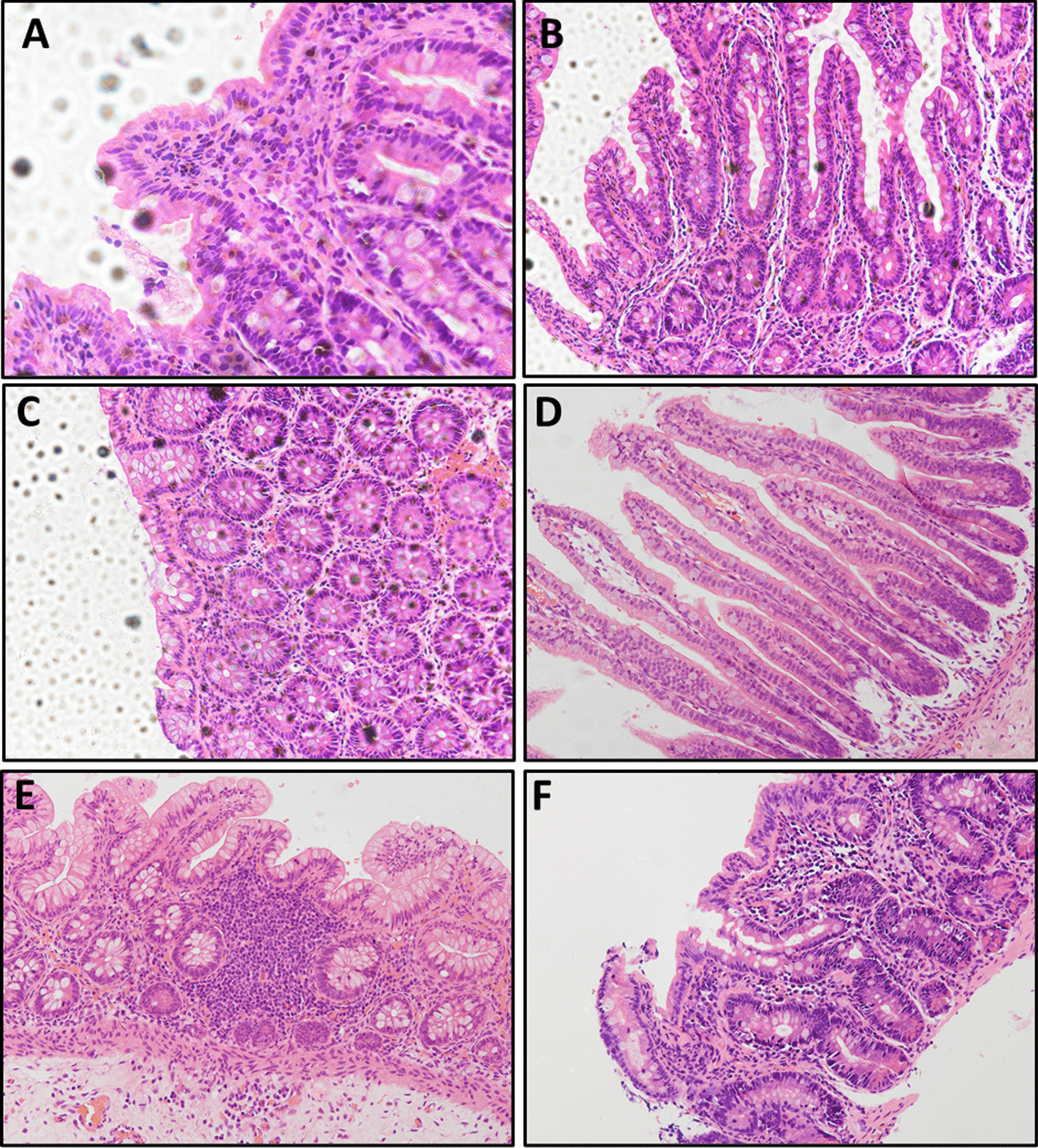
H&E staining of intestinal biopsies from Patients 2–4. **A** Villus flattening, disorganized epithelium in the duodenum biopsy from Patient 2 (400×). **B** Villus atrophy with crowded epithelial cells in the terminal ileal mucosal biopsy from Patient 2 (200×). **C** Chronic inflammation; villous atrophy in the colon from Patient 2 (200×). **D** Normal villous height in the ileum from Patient 3 (200×). **E** Hyperemia, edema, and lymphocyte aggregates in the colon from Patient 3 (200×). **F** Hyperemia, edema, and prominent lymphocyte infiltration; villous blunting in duodenum from Patient 4 (200×)

Apart from an elevated number of white blood cells and metabolic acidosis, no other abnormal finding was detected in liver function, renal function, immunoglobulin levels, thyroid hormones, autoimmune antibodies, or infectious etiologies when the child was admitted. A gastrointestinal endoscopy was carried out at 43 days old but no obvious gross manifestations were noticed (Fig. 1C, D). The H&E staining of biopsies showed villous atrophy and crowded epithelial cells (Fig. 4A–C).

Fasting resulted in transient relief of diarrhea symptoms, but the stool output increased to 600 g/day after intake of an amino acid-based formula (AAF). The patient underwent 1 episode of sepsis during a 1-month stay in our hospital. The child has been on partial PN for more than a year after discharge, but weighed only 3.3 kg on his first birthday.

##### Patient 3

**Fig. 5:**
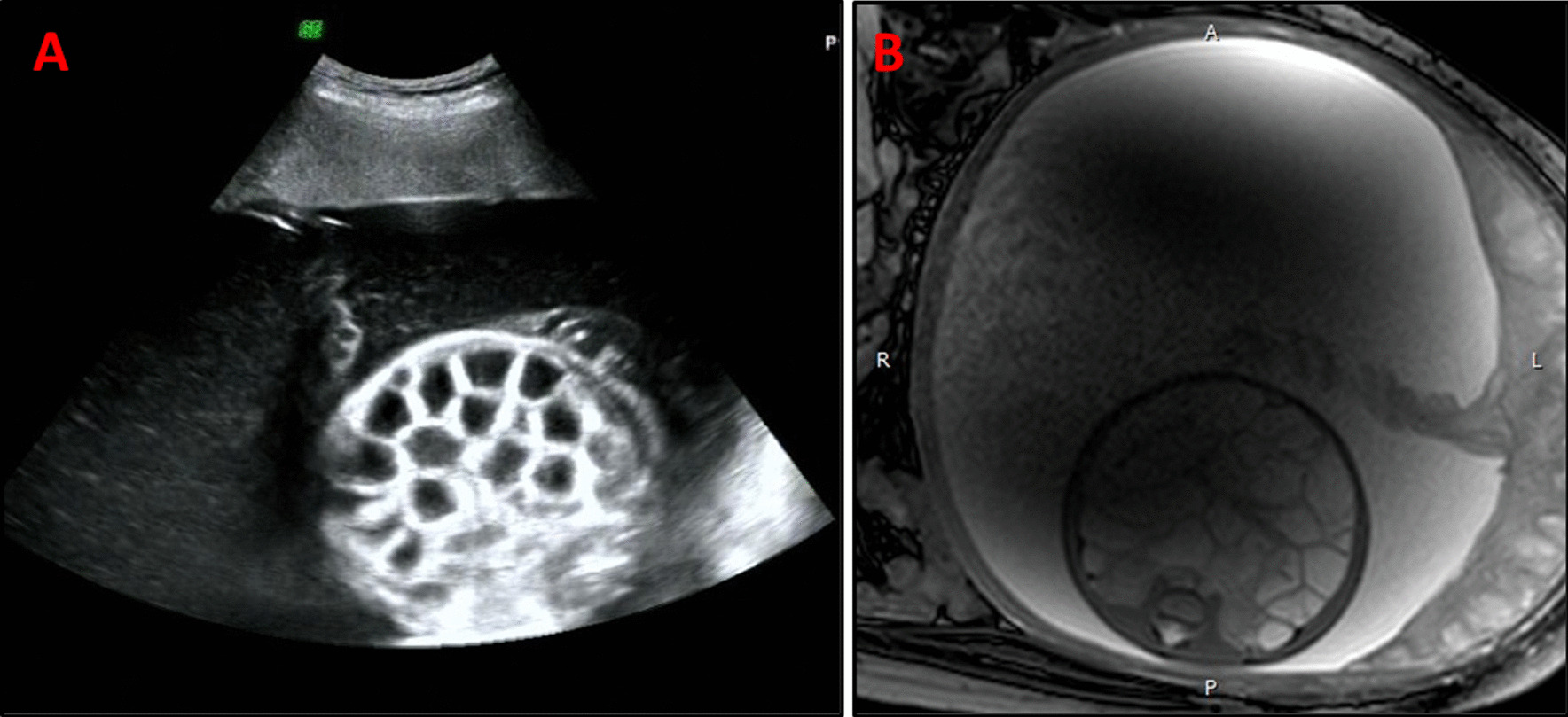
Prenatal imaging of Patient 3. **A** Dilated loops of fetal bowel and polyhydramnios were visible by ultrasound at 24 weeks’ gestation. **B** MRI scanning confirmed the presence of polyhydramnios and fetal diffuse intestinal dilation at 26 weeks’ gestation

A 3-month-old male infant was admitted to our division for failure to thrive, and with intractable diarrhea. The patient was born at 35^+5^ weeks gestation, with a weight of 3020 g, via vaginal delivery in our hospital. Prenatal imaging showed dilated loops of bowel and polyhydramnios at 24–26 weeks’ gestation (Fig. 5A, B) and Grade I meconium-stained amniotic fluid was noted at birth. There was no correlative family history. The infant had 3–5 stools daily but still presented with abdominal distention after birth. Intestinal stenosis was suspected after he underwent barium enema imaging. A bowel resection and Bishop Koop ileostomy were performed in the distant ileum on day of life (DOL) 4. The H&E staining found inflammatory changes without villous atrophy in the ileum or colon (Fig. 4D, E). He suffered from recurrent watery diarrhea and retardation after being discharged from the Department of Surgery. His body weight was just 3.5 kg (Z score − 4.12) and the length was 55 cm (Z score − 3.18) at 3 months old when he was transferred to our division for failure to thrive.

At the age of 3 months, when he was referred to our division, the patient’s laboratory tests revealed typical hypochloremic metabolic alkalosis, hyponatremia, and hypokalemia. Other findings were normal, including liver function, renal function, fecal pathogens, immunoglobulin levels, thyroid hormones, and complete blood cell count.

The infant was given extensively hydrolyzed formula by nasogastric intermittent feeding and supplemented with potassium, which alleviated the diarrhea and rectified the alkalosis and potassium deficiency as well. He was able to sit independently and his body weight had increased to 7.5 kg (Z score − 0.92) at 7 months old.

##### Patient 4

A 42-day-old boy was admitted to the NICU for moderate dehydration and lethargy. He was born with a birth weight of 3300 g at 40^+3^ weeks gestation by vaginal delivery. His parents and 11-year-old sister were healthy. At 28 days after birth, he developed diarrhea. His stools were watery and evacuated more than 10 times per day.

The initial test results showed elevated levels of alanine transaminase (ALT), metabolic acidosis, and hypokalemia. The fecal pathogen tests were negative. The patient was treated with different antibiotics (cefmetazole, cefepime + metronidazole, vancomycin + meropenem) in accordance with the infectious indications. Other treatments included smectite, racecadotril, hepatic protection, and PN. However, no significant improvement was achieved after a month of treatment. The patient was then referred to the division of pediatric gastroenterology and nutrition, and underwent endoscopic examination (Fig. 1E, F). The results of H&E staining showed that villus blunting was present in both duodenal (Fig. 4F) and ileal biopsies. TEM analysis did not show any typical inclusion bodies in terminal ileal biopsies (Fig. 3E, F).

At the age of 2 months, the patient received mechanical ventilation for 3 days and anti-epileptic drugs due to recurrent seizures, presenting with repeated muscular spasms on the face and in the limbs. He was discharged with a weight of 4.1 kg when both seizures and sepsis were well controlled.

#### Genetic analysis

The genetic analyses of the 4 patients are showed in Table 3.

**Table 3:** Genetic analysis of four patients with neonatal-onset watery diarrhea

	Patient 1	Patient 2	Patient 3	Patient 4
MIM	185,535	185,535	214,700	251,850
Gene	*EPCAM*	*EPCAM*	*SLC26A3*	*MYO5B*
Position (Hg19)	Chr2:	Chr2:	Chr7:	Chr18:
	47,600,715	47,600,621	107,415,299	47,500,736
	47,596,623–47,613,791	47,607,073	107,434,187	47,405,401
Transcripts	NM_002354.2	NM_002354.2	NM_000111.2	NM_001080467.2
*Variants (cDNA)*				
Variants1	c.184 + 6T > G	c.96 C > A	c.1696 C > T	c.1306 G > T
Variants2	Exon 1–9 large deletion	c.823delG	c.269_270dupAA	c.3190 C > T
*Variants (protein)*				
Variants1	Splice-site change	p.Y32*	p.R566*	p.V436F
Variants2	Exon 1–9 large deletion	p.V275Wfs*2	p.G91Kfs*3	p.R1064*
*Consequence*				
Variants1	Splicing mutation	Nonsense	Nonsense	Missense
Variants2	17.168 kb deletion	Frameshift	Frameshift	Nonsense
*Exon/intron*				
Variants1	Intron 2	Exon 2	Exon 16	Exon 10
Variants2	Exon 1–9 large deletion	Exon 7	Exon 3	Exon 24
*Parental origin*				
Variants1	Maternal	Maternal	Paternal	Paternal
Variants2	Paternal	Paternal	Maternal	Maternal
*Pathogenicity*				
Variants1	Pathogenic	Likely pathogenic	Pathogenic	Likely pathogenic
Variants2	Pathogenic	Likely pathogenic	Pathogenic	Likely pathogenic

##### Patient 1

The results of whole-exome sequencing (WES) confirmed the presence of novel complex mutations in the *EPCAM* gene (NM_002354) in Patient 1. The patient’s mother was a heterozygous carrier of a mutation (c.184 + 6T > G) in intron 2 (NM_002354.2), which was classified as ‘pathogenic’. A large 17.168 kb deletion was predicted in his father’s chromosome 2 (chr2:47596623–47613791) using the eXome-Hidden Markov Model (XHMM) program. The patient inherited both genetic abnormalities from his asymptomatic parents. No mutations were detected when the sister’s DNA was sequenced.

##### Patient 2

Novel complex heterozygous mutations in exon 2 (c.96 C > A; p.Y32*) and in exon 7 (c.823delG; p.V275W fs*2) of the *EPCAM* gene were detected in Patient 2. The patient inherited the c.96 C > A mutation from his mother and the c.823delG mutation from his father. Both variants were classified as ‘likely pathogenic’ based on the American College of Medical Genetics and Genomics/Association for Molecular Pathology (ACMG/AMP) 2015 guidelines.

##### Patient 3

The sequencing results for Patient 3 confirmed the presence of complex heterozygous mutations (c.269_270 dupAA and c.1696 C > T) in the *SLC26A3* gene. The patient’s mother was a heterozygous carrier of the c.269_270 dupAA mutation (p.G91Kfs*3), whereas the c.1696 C > T mutation (p.R566*) was carried by the father. Both variants were classified as ‘pathogenic’ according to the AMCG/AMP 2015 guidelines.

##### Patient 4

Novel *MYO5B* variants were identified in Patient 4. The compound heterozygous mutations were c.1306 G > T (p.V436F), which was inherited from the father, and c.3190 C > T (p.R1064*), which came from the mother. Both variants have not been previously described, either in the Human Gene Mutation Database (HMGD) or in the PubMed database. The nonsense variant c.3190 C > T was classified as ‘likely pathogenic’ with PVS1 (loss of function) and PM2 (absent from controls) evidence according to the AMCG/AMP 2015 guidelines. The other novel missense variant was evaluated to have PM2, PM1 (mutation hotspot), and PP3 (in silico evidence) pathogenic evidence, and was predicted to be “deleterious”, “probably damaging”, or “disease_causing” by the Sorting Intolerant from Tolerant (SIFT), PolyPhen-2, and Mutation Taster algorithms, respectively.

### Review of mutations in East Asia

We reviewed the mutations in *SLC26A3*, *EPCAM*, and *MYO5B* reported in English or Chinese language in East Asia (Table 4). There were 34 *SLC26A3* mutations detected according to the literatures. Among them, the c.269_270 dupAA (p.G91Kfs*3) mutation in exon 3 is the most frequent genetic abnormality found in 7 CCD patients in China. In contrast, reports from Korea and Japan showed the c.2063-1G > T mutation in intron 18 was the most common alteration, which existed in 16 cases. Most patients with CCD carried mutations in exon 3–5, exon 9 and intron 18. To date, the *EPCAM* and *MYO5B* variants have rarely been reported in East Asia. Nine *EPCAM* mutations have been identified in patients with CTE, 3 of which were located in exon 3.

**Table 4:** Reported mutations in *SLC26A3, EPCAM,* and *MYO5B* in East Asia

Gene	Exon/intron	Mutation type	cDNA variant	protein variant	Patients (n)	Region	Reference
*SLC26A3*	Exon 3	Missense	c.239G > A	p.G80D	1	China	Zhang, et al. [3]
	Exon 3	Insertion	c.268-269insAA/ c.269-270dupAA	p.G91Kfs*3	7	China	This study; Liu et al., Lin et al., Wang et al. [4–6]
	Exon 4	Missense	c.358G > T	p.G120C	2	Japan	Konishi et al. [7]
	Exon 4	Missense	c.358G > A	p.G120S	1	Japan	Konishi et al. [7]
	Exon 4	Deletion	c.354delC	p.F119Sfs*15	1	Japan	Matsunoshita et al. [8]
	Exon 4	Missense	c.272G > T	p.G91V	1	Korea	Bhardwaj et al. [9]
	Exon 4	Missense	c.358G > A	p.G120S	1	China	Li et al. [10]
	Exon 5	Missense	c.392C > G	p.P131R	1	Japan	Konishi et al. [7]
	Exon 5	Missense	c.392C > T	p.P131A	1	Japan	Konishi et al., Fuwa et al. [7, 11]
	Exon 5	Missense	c.401G > A	p.S134N	1	Korea	Hong et al.[12]
	Exon 5	Missense	c.392C > T	p.P131L	2	Korea	Hong et al. [12]
	Exon 5	Missense	c.525G > C	p.R175S	1	Korea	Lee et al.[13]
	Exon 6	Missense	c.634G > T	p.G212C	1	China	Li et al. [10]
	Exon 7	Missense	c.877G > A	p.E293K	1	Japan	Matsunoshita et al. [8]
	Exon 9	Insertion	c.1007-1008insT	p.F336Ffs*34	5	Japan	Konishi et al. [7, 8]
	Exon 9	Missense	c.1043T > A	p.M348K	1	Japan	Konishi et al. [7]
	Exon 9	Missense	c.1039G > A	p.A347T	1	China	Wu et al. [14]
	Exon 10	Missense	c.1198T > C	p.S400P	1	Japan	Konishi et al. [7]
	Exon 11	Missense	c.1299G > A	p.A433A	ND	Japan	Lechner et al. [15]
	Exon 12	Deletion	c.1342-1343del	p.L448Kfs*9	4	Japan	Makela et al., Konishi et al. [7, 16]
	Exon 12	Missense	c.1387C > T	p.R463X	1	China	Guo et al. [17]
	Exon 13	Missense	c.1487T > G	p.L496R	3	China	Makela et al. [16]
	Exon 13	Missense	c.1483C > A	p.Q495K	1	China	Guo et al. [17]
	Exon 15	Missense	c.1631T > A	p.I544N	2	China	Lei et al., Song et al. [18, 19]
	Exon 15	Missense	c.1644C > G	p.N548K	1	Japan	Konishi et al. [7]
	Exon 15	Missense	c.1661G > A	p.R554Q	ND	Japan	Lechner et al. [15]
	Exon 16	Nonsense	c.1696C > T	p.R566*	1	China	This study
	Exon 18	Missense	c.2048T > A	p.I683N	1	China	Liu et al. [4]
	Intron 6	Splice-site change	c.735 + 4_735 + 7del	Intron donor site GT loss	1	China	Lin et al. [5]
	Intron 7	Splice-site change	c.888 + 1G > A	Intron donor site GT loss	2	Japan	Konishi et al. [7]
	Intron 12	Splice-site change	c.1407 + 3A > C	Intron donor site GT loss	1	Korea	Hong et al. [12]
	Intron 15	Splice-site change	c.1677 + 1G > C	Intron donor site GT loss	1	Japan	Konishi et al. [7]
	Intron 18	Splice-site change	c.2063-1G > T	Intron acceptor site AG loss	7	Japan	Konishi et al., Fuwa et al. [7, 11]
					9	Korea	Hong et al., Lee et al. [12, 13]
	Intron 6 to 8	Deletion	3.5 kb deletion	Exon 7 to 8 deletion	2	Japan	Makela et al. [16]
*EPCAM*	Exon 2	Nonsense	c.96C > A	p.Y32*	1	China	This study
	Exon 3	Missense	c.316A > T	p.K106X	2	Korean	Ko et al. [20]
	Exon 3	Missense	c.307G > A	p.G103R	1	China	Tang et al. [21]
	Exon 3	Missense	c.412C > T	p.R138X	1	China	Yuan et al. [22]
	Exon 7	Deletion	c.823delG	p.V275Wfs*2	1	China	This study
	Intron 2	Splice-site change	c.184 + 6T > G	Splicing mutation	1	China	This study
	Intron 5	Splice-site change	c.491 + 1G > A	Splicing mutation	2	Korean	Ko et al. [20]
	Exon 1–9	Large deletion	Exon 1–9 deletion	Exon 1–9 deletion	1	China	This study
	Exon 2–5	Large deletion	Exon 2–5 deletion	Exon 2–5 deletion	1	China	Tang et al. [21]
*MYO5B*	Exon 4	Missense	c.445C > T	p.Q149X	2 (sibling)	Taiwan, China	Chen et al. [23]
	Exon 9	Missense	c.1021C > T	p.Q341X	2 (sibling)	Taiwan, China	Chen et al. [23]
	Exon 10	Missense	c.1306G > T	p.V436F	1	China	This study
	Exon 16	Missense	c.1966C > T	p.R656C	1	China	Cheng et al. [24]
	Exon 21	Deletion	c.2729_2731delC	p.R911Afs916*	1	China	Mao et al. [25]
	Exon 24	Nonsense	c.3190C > T	p.R1064*	1	China	This study
	Intron 3	Splice-site change	c.310 + 2Tdup	Splicing mutation	1	China	Cheng et al. [24]
	Intron 37	Splice-site change	IVS37-1G > C	Splicing mutation	1	China	Mao et al. [25]

*ND* no data

## Discussion

The diagnosis and management of neonatal-onset diarrhea are particularly challenging. Common etiologies include gastrointestinal food allergies, infections, congenital enterocyte defects, enteroendocrine cell dysfunction, and immune dysregulation-associated enteropathy. Cases that present with intractable early-onset diarrhea, that can potentially lead to intestinal failure, should be suspected as congenital genetic disorders [1]. With this in mind, advances in genomic medicine can be used to help physicians identify the precise diagnosis for these disorders.

### Congenital tufting enteropathy

CTE is a rare autosomal recessive enteropathy, presenting with intractable neonatal-onset diarrhea, severe malnutrition, and intestinal failure [26–28]. The incidence of CTE is estimated to be approximately 1 in 50,000–100,000 live births in Western Europe [29]. When the intestinal tissues of CTE patients were examined by histology, the results showed villous atrophy, abnormalities of the basement membrane, and “tufts” with closely packed epithelial enterocytes at the villus tips [27, 30]. However, the typical tufts revealed by H&E staining are difficult to detect in the early months of life [29]. Similarly, these prototypical tufts were evident in Patient 1 but not in Patient 2 in this study.

Variants in the *EPCAM* gene (which encodes an epithelial cell adhesion molecule) and the serine peptidase inhibitor Kunitz type 2 (*SPINT2*) gene were found to be associated with CTE [21, 30, 31]. Loss of *EPCAM* expression disrupts tight junctions, leading to increased permeability of the intestinal barrier and decreased ion transport, resulting in diarrhea [28, 32, 33]. The c.498 insC mutation in exon 5 of EPCAM is the most frequent homozygous mutation in the Middle East [21, 34]. Mutations in exon 3 are much more wide-spread and variable across populations [21], as well as East Asia.

Phenotypes can differ greatly among patients with CTE. Some cases of CTE have been associated with multiple arthritis [20, 35] or malformations, including photophobia, keratitis, cataracts, choanal atresia [36], and cardiomyopathy [37].

It has been reported that patients with CTE may have a greater than 92% chance of long-term survival and 75% can be weaned off parenteral nutrition (PN) by 25 years old [27]. Lemale et al. [38] found that patients who were weaned off PN showed improvements in their histological lesions over time.

The first Chinese CTE case was reported in 2018 by Tang et al. [21]. In this study, the novel *EPCAM* mutations seen in the two unrelated children with CTE provide further evidence that the *EPCAM* gene is involved in the pathogenesis of CTE. WES enabled us to identify CTE earlier in Patient 2 whose histology didn’t detect typical tufts. We hypothesize that CTE prevalence may be underestimated in China to date.

### Congenital chloride diarrhea

CCD is inherited in an autosomal recessive fashion and manifests in the form of large-volume, acidic, watery, profuse diarrhea that starts in the fetal stage, with concomitant hypochloremic metabolic alkalosis [7, 39]. In general, the prenatal examination tends to reveal fetal diffuse intestinal dilation and polyhydramnios caused by intrauterine diarrhea [7, 39], which is very analogous to intestinal obstruction. A high fecal chloride level (> 90 mmol/L) can be predictive in the initial diagnosis. The management of CCD includes chloride supplementation for life and fluid supplementation when the patient’s stool output increases.

The pathogenic profile of CCD is associated with monogenic mutations in the solute carrier *SLC26A3* gene [7]. This gene encodes a coupled chloride/bicarbonate (Cl–/HCO3–) exchanger, which functions in conjunction with sodium/hydrogen exchangers (Na + /H + exchangers, NHE), and is required for electroneutral sodium absorption, particularly in the ileum and colon. *SLC26A3* mutations result in a loss of intestinal sodium-driven fluid absorption, leading to distal ileal chloride loss, bicarbonate retention, and hypochloremic metabolic alkalosis. Hypokalemia is mainly attributed to the activation of the renin/angiotensin/aldosterone system.

Konishi et al. [7] reported a multi-institutional survey on patients in Japan with confirmed CCD. Most of the patients (62%) had less than 5 stools daily, and all of them had fewer after infancy. Only 38% of patients needed long-term sodium, potassium, and chloride supplementation. Their neurodevelopment was generally good except for one patient. Another patient had nephrocalcinosis, and three patients (23%) presented with mild chronic kidney disease. Homozygous or compound heterozygous *SLC26A3* variants were identified in 13 of 14 patients (93%).

We noticed that Patient 3 was possibly misdiagnosed with intestinal stenosis before 6 months old. It is particularly important to be aware that CCD could also be suspected in cases with prenatal polyhydramnios and extensive intestinal dilation, so as to avoid unnecessary surgeries as a result of misdiagnosis as intestinal obstruction. The c.269_270 dupAA (p.G91Kfs*3) mutation has also been described as c.269_270 insAA or c.268_269 insAA, which is the most frequent mutation reported previously in 6 patients in China (20–24). A newly described variant, c.3190 C > T (p.R1064*), has been released on https://www.ncbi.nlm.nih.gov/snp (rs1018933248) by other researchers on Jul 13, 2019.

### Microvillus inclusion disease

MVID is a rare autosomal recessive diarrheal disease, associated with defects in epithelial trafficking within the microvilli, that results in dramatic diarrhea leading to metabolic acidosis, electrolyte abnormalities, and failure to thrive [1, 40]. The pathological characteristics of the disease include villus atrophy, blunted or absent microvilli, and inclusion bodies in the enterocytes [40]. It is important to note that the identification of inclusion bodies requires repeated TEM analyses and the frequency of occurrence could be very low. Some individuals with MVID did not show typical microvillus inclusions but did exhibit other enterocyte abnormalities [41, 42]. Patients with MVID require long-term PN and may eventually be referred for intestinal transplantation [40].

Various mutations in *MYO5B*, *STXBP2*, and *STX3* are reported to be associated with MVID [40, 41]. The *MYO5B* gene encodes the actin-based motor protein, myosin VB. Myosin VB, RAB11A, and RAB8A associate with apical recycling endosomes (AREs) and play a vital role in the development of epithelial polarity. Impairments in RAB11A and RAB8A-dependent AREs in the small bowel have been demonstrated in MVID by immunostaining tests. Both myosin VB and RAB11A are mislocalized in MVID enterocytes. Defects in AREs result in microvillus atrophy and the intracellular retention of enzymes and transporters required for nutrient and ion absorption. These enterocytes with dysfunctional microvilli are unable to absorb nutrients and fluids, leading to severe diarrhea in individuals with MVID.

In this study, Patient 4 presented with intractable watery diarrhea, metabolic acidosis, electrolyte abnormalities, and liver dysfunction, but inclusion bodies were not detected by TEM. The use of WES enabled us to recognize the etiology of the disease as MVID in this patient.

### Genetic testing

The increased use of genetic sequencing can allow for prompt recognition of the etiology underpinning monogenic diarrhea and thus guide diagnosis-directed treatment. As Thiagarajah et al. [2] has suggested, targeted Sanger sequencing should be considered for rapid diagnosis in cases where the clinical evaluation is strongly suggestive of a particular disorder, such as the presence of characteristic epithelial tufts and microvillus inclusion in biopsy slides. In cases where a diagnosis based on clinical evaluation is unclear, it is necessary to perform WES. Whole-genome sequencing (WGS) or RNA sequencing should also be considered in some highly suspected cases when WES fails to detect genetic variants [2]. In this study, we employed WES to clarify the etiologies of all four cases. Firstly, it has been reported that the prototypical epithelial tufts or microvillus inclusions could be difficult to detect by histology in some individuals with CTE or MVID [29, 41, 42]. In line with this drawback, the appearances of the intestinal epithelia on H&E staining or TEM performed on endoscopic biopsies from Patient 2, and Patient 4 were not suggestive of any disease. Secondly, CCD is not easily differentiated from Bartter syndrome and some other disorders presented as pseudo-Bartter syndrome (for example, cystic fibrosis, Pendred syndrome, mineralocorticoid excess syndrome) [43], and the diarrhea was not severe in Patient 3.

After a breakthrough in the clinical utility of NGS technology, more and more cases are being confirmed as the congenital diarrheal disorders. However, these rare etiologies have not been well recognized by pediatricians and pathologists in China. Hopefully, these reports of CTE, CCD, and MVID will increase the spectrum of genotype–phenotype correlations and help more pediatricians to identify such disorders.

## Conclusions

The etiologies of patients with neonatal-onset watery diarrhea could be difficultly diagnosed on the basis of clinical manifestation and histology assessment, especially in their early days of life. With this study, we described the clinical manifestation and genetic analyses of 4 patients with neonatal-onset watery diarrhea. Novel compound mutations were identified respectively in the *EPCAM* gene of two children and *MYO5B* gene of one other boy. Furthermore, we reviewed the genetic variants of *SLC26A3, EPCAM, and MYO5B* reported in East Asia. It reveals that c.269_270 dupAA (p.G91Kfs*3) is the most frequent *SLC26A3* mutation in China. *EPCAM* and *MYO5B* genetic variants were only sporadically reported in East Asia. Early diagnosis based on genetic analysis is essential to get better prognosis, leading to improved treatment. Future research should explore the functional effects of the novel mutations identified in this study.

## Material and methods

### Biological samples

We retrospectively reviewed the clinical data of four children at Xinhua Hospital Affiliated to Shanghai Jiao Tong University School of Medicine. The biopsy specimens from colonoscopy or surgery were fixed in formaldehyde for H&E staining or in glutaraldehyde for electron microscopy. Paraffin-embedded sections of tissue specimens were mounted on glass slides and H&E staining was performed in the Department of Pathology of our hospital. The surgery biopsies of Patient 1 were assessed by TEM at Shanghai Jiao Tong University School of Medicine. The TEM assessments of tissues from Patient 2 and Patient 4 were carried out by KingMed Diagnostics Corporation (Shanghai, China).

### Exome sequencing

#### Patient 1

Genomic DNA was extracted from peripheral blood (processed with EDTA anticoagulant) acquired from Patient 1 and his family members, using PureLink Genomic DNA Mini Kit (Invitrogen, Thermo Fisher Scientific, MA, USA). WES was conducted using the Illumina HiSeq4000 platform at Berry Genomics (Beijing, China). Exome capture was performed using the xGen Exome research panel v1.0 (IDT, Iowa, USA). The exome design covered 97.9% of the coding regions of the 19,396 genes analyzed. The mean coverage was 154.0 reads for the patient’s sample, and 97.3% of the target region was covered by at least 10 reads.

The sequencing data were analyzed by the Shanghai Institute for Pediatric Research. Sequence data were aligned to the reference sequences of the human genome assembly GRCh37 (hg19) using the Burrows-Wheeler Aligner (BWA) algorithm, BWA-MEM version 0.7.12. Base quality score recalibration (BQSR), indel realignment, and duplication removal were performed using the Genome Analysis Toolkit (GATK) version 3.3 and both single nucleotide variants (SNV) and indels were detected according to GATK best practice recommendations. Copy number variation (CNV) was analyzed using the eXome-Hidden Markov Model (XHMM) program, version 1.0. The SnpEff (version 4.2) and SnpSift (version 4.2) programs were used to select variants based on the following databases: gnomAD, OMIM, HGMD, ClinVar, REVEL, splicing mutation-Human Splicing Finder (v3.1), MaxEntScan, NNSplice, and local patient databases. Variant interpretation was based on the following guidelines and recommendations: a joint consensus recommendation of the ACMG and AMP [44]; guidelines from the ClinGen Sequence Variant Interpretation Working Group (https://clinicalgenome.org/); and the Association for Clinical Genomic Science (ACGS) Guidelines for Variant Classification (https://www.acgs.uk.com/). Variants that were present at a frequency of > 1% in the 1000 Genomes Project (http://www.1000genomes.org/), ExAC (http://exac.broadinstitute.org/), or in the Exome Variant Server (http://evs.gs.washington.edu/EVS/) at a frequency of > 5% in the Inhouse database were excluded.

#### Patient 2

WES of DNA from Patient 2 and his parents was performed and analyzed by Jiang Jian Medical Testing Corporation (Guangzhou, China). Genomic DNA was extracted from peripheral blood incubated with EDTA anticoagulant using the Solpure Blood DNA kit (Magen, Guangzhou, China) according to the manufacturer’s instructions. The genomic DNA was then fragmented using a Q800R Sonicator (Qsonica, Newtown, CT, USA) to generate 300–500 bp insert fragments. The paired-end libraries were prepared following the Illumina protocol. Custom designed NimbleGen SeqCap probes (Roche NimbleGen, Madison, Wis) were used for in-solution hybridization to enrich for target sequences. Enriched DNA samples were indexed and sequenced on a NextSeq500 sequencer (Illumina, San Diego, CA, USA). The samples were sequenced at a coverage of 340+/− 190×. The coverage of the target region was 99.9% with at least 10 reads.

The primary data were provided in fastq format, after image analysis and base calling was conducted using the Illumina Pipeline. The data were filtered to generate ‘clean reads’ by removing adapters and low-quality reads (Q20). Sequencing reads were mapped to the reference human genome assembly version hg19 (http://genome.ucsc.edu/). Nucleotide changes observed by aligned reads were called and reviewed by using NextGENe software (SoftGenetics, State College, Pa). The sequence variants were annotated using various databases, including 1000 Genomes, dbSNP, GnomAD, Clinvar, HGMD, and OMIM. The assessment of mutation pathogenicity prediction was performed according to the ACMG guidelines.

#### Patient 3

WES and data analysis were performed for the patient and parents as described for Patient 1. The mean coverage was 157.6, 183.9, and 171.1 reads for the patient, father, and mother respectively, and 98.5–98.7% of the target region was covered by at least 10 reads.

#### Patient 4

The WES sequencing was carried out for the patient and Sanger sequencing was performed on DNA from the parents for verification, by Kangso Medical Inspection (Beijing, China).

The variants were selected based on the 1000Genomes and dbSNP databases. The assessment of mutation pathogenicity prediction was based on a joint consensus recommendation of the ACMG and AMP.

## Data Availability

The data are available on request from the first author.

## Abbreviations

NGS: Next-generation sequencing
*EPCAM*: Epithelial cell adhesion molecule
CCD: Congenital chloride diarrhea
*SLC26A3*: Solute carrier family 26 member 3
MVID: Microvillus inclusion disease
*MYO5B*: Myosin VB
WES: Whole-exome sequencing
CODEs: Congenital diarrhea and enteropathies
IF: Intestinal failure
CTE: Congenital tufting enteropathy
IPEX: Immune dysregulation, polyendocrinopathy, enteropathy, X-linked
H&E: Hematoxylin–eosin
TEM: Transmission electron microscopy
FMT: Fecal microbiota transplant
PN: Parenteral nutrition
NICU: Neonatal intensive care unit
AAF: Amino acid-based formula
DOL: Day of life
ALT: Alanine transaminase
ACMG: American College of Medical Genetics and Genomics
AMP: Association for molecular pathology
HMGD: Human gene mutation database
*SPINT2*: Serine peptidase inhibitor Kunitz type 2
AREs: Apical recycling endosomes
OMIM: Online Mendelian inheritance in man
